# The possible role of cross-reactive dengue virus antibodies in Zika virus pathogenesis

**DOI:** 10.1371/journal.ppat.1007640

**Published:** 2019-04-18

**Authors:** Thomas Langerak, Noreen Mumtaz, Vera I. Tolk, Eric C. M. van Gorp, Byron E. Martina, Barry Rockx, Marion P. G. Koopmans

**Affiliations:** Department of Viroscience, Erasmus Medical Center, Rotterdam, the Netherlands; University of Alberta, CANADA

## Abstract

Zika virus (ZIKV) has been known for decades to circulate in Africa and Asia. However, major complications of a ZIKV infection have recently become apparent for reasons that are still not fully elucidated. One of the hypotheses for the seemingly increased pathogenicity of ZIKV is that cross-reactive dengue antibodies can enhance a ZIKV infection through the principle of antibody-dependent enhancement (ADE). Recently, ADE in ZIKV infection has been studied, but conclusive evidence for the clinical importance of this principle in a ZIKV infection is lacking. Conversely, the widespread circulation of ZIKV in dengue virus (DENV)-endemic regions raises new questions about the potential contribution of ZIKV antibodies to DENV ADE. In this review, we summarize the results of the evidence to date and elaborate on other possible detrimental effects of cross-reactive flavivirus antibodies, both for ZIKV infection and the risk of ZIKV-related congenital anomalies, DENV infection, and dengue hemorrhagic fever.

## Introduction

Zika virus (ZIKV) is an arthropod-borne flavivirus in the family *Flaviviridae*, which includes several other arthropod-borne viruses of clinical importance, such as dengue virus (DENV), West Nile virus (WNV), and yellow fever virus (YFV) [1]. ZIKV is a positive-sense single-stranded enveloped RNA virus. The genome encodes a polyprotein, which is processed into three structural proteins (the capsid [C], premembrane [prM], and the envelope [E] protein) and seven nonstructural proteins (NS1, NS2A, NS2B, NS3, NS4A, NS4B, NS5) [2]. Until 2006, literature was limited, and no large outbreaks of ZIKV were reported [3]. This changed in 2007 with the first report of a major outbreak of ZIKV on the island of Yap in Micronesia, followed by another large outbreak in French Polynesia in 2013 [4, 5]. In May 2015, ZIKV infection was reported in Brazil, which was the first report of locally acquired ZIKV in South America, and heralded an unprecedented outbreak across the Americas and the Caribbean. Phylogenetic studies estimate that between late 2013 and early 2014, ZIKV was introduced from the Pacific Islands into the northeast of Brazil, where it spread to other regions and countries [6–8]. Several months after the start of the 2015–2016 ZIKV outbreak, unusually high numbers of Guillain–Barré syndrome (GBS) cases were observed in adults and of microcephaly cases in fetuses and newborn infants [9]. For the congenital abnormalities, it became clear that microcephaly constituted the proverbial tip of the iceberg, and since then several other severe abnormalities have been associated with a congenital ZIKV infection, such as lissencephaly, ventriculomegaly, and ocular abnormalities [10–13].

Relatively early in the epidemic, the causal relationship between ZIKV and congenital abnormalities was established [14, 15]. A burning question has been why these congenital abnormalities were only seen in the recent ZIKV outbreaks in the Americas. Could preexisting immunity to other flaviviruses explain this phenomenon? Recently, a considerable amount of research has been performed to investigate whether antibody-dependent enhancement (ADE) of ZIKV can explain the seemingly increased pathogenicity of ZIKV. The rationale behind this consideration is that, in the closely related DENV, ADE plays an important role in the increased risk of developing severe symptoms during secondary DENV infection [16]. In this review, we discuss the evidence for ADE of ZIKV infection. Because of differences in tissue tropism of ZIKV and DENV, it should be taken into account that clinical presentations of infection enhancement by cross-reactive antibodies can differ between these viruses [17, 18]. Therefore, we elaborate on a specific route by which cross-reactive dengue antibodies could have a detrimental effect in ZIKV infection, namely by facilitating vertical transmission of ZIKV from mother to fetus during pregnancy. Finally, we discuss the potential implications of cocirculation of ZIKV and DENV for the problem of DENV ADE.

## The placental barrier

The most notorious complications of a ZIKV infection are the severe congenital abnormalities it can cause. In order to discuss whether and how cross-reactive dengue antibodies can play a role in these complications, it is important to understand how ZIKV can reach the fetus during pregnancy. One way for ZIKV to infect the fetus during pregnancy is through transplacental transmission. The placenta is an important protective barrier against pathogens for the fetus. The human placenta consists of many chorionic villi; the anchoring chorionic villi are attached to the mucosal lining of the uterus (decidua), whereas the floating chorionic villi float around in maternal blood in the intervillous space, where gas and nutrient exchanges take place. The chorionic villi are lined by two types of trophoblasts: an outer layer of terminally differentiated multinuclear syncytiotrophoblasts (STBs) and mononuclear cytotrophoblasts (CTBs), which are situated underneath the STB layer and can differentiate into STBs or extravillous trophoblasts (EVTs) that infiltrate the decidua in anchoring villi. The STB layer is important for protection against pathogens and has previously been demonstrated to be resistant to infection from many pathogens, including cytomegalovirus (CMV), *Toxoplasma gondii*, and *Listeria monocytogenes* [19–21]. CTBs and EVTs, on the other hand, are susceptible to some pathogens, including *T*. *gondii* and CMV [21, 22]. To enter the villus core and reach the fetal circulation, pathogens either have to cross the STB layer in floating villi or infect EVTs in anchoring villi. Recently, how and when ZIKV can cross the placenta have been investigated in experimental studies and clinical observations.

## First-trimester placentas seem most permissive for ZIKV

Analysis of placentas of women with a suspected ZIKV infection showed that the relative level of ZIKV RNA was 25-fold higher in first-trimester placentas compared with second- and third-trimester placentas [23]. In placentas of women infected with ZIKV, using in situ hybridization (ISH), ZIKV was consistently identified in only the Hofbauer cells (HBCs), which are the placental macrophages that are located in the chorionic villus core, and not in CTBs or STBs [23, 24]. In addition, multiple in vitro studies that were performed with primary placental cells isolated from early- and late-pregnancy placental explants found that ZIKV replicates in CTBs isolated from first-trimester placenta explants [25–28], whereas in CTBs isolated from term placenta explants, only low replication of ZIKV was observed [27, 29]. It was also demonstrated that STBs obtained from term placentas were resistant to ZIKV, possibly because of the production of type III interferons [30]. These observations suggest that the placenta is more susceptible to ZIKV infection during the first trimester of pregnancy than during the second and third trimesters of pregnancy. In contrast to the differential sensitivity of CTBs and STBs from placentas in different stages of pregnancy, many of the above-mentioned studies found similar levels of replication of ZIKV in HBCs isolated from both early- and full-term placentas, suggesting that these cells can possibly serve as a replication reservoir for ZIKV once the virus has entered the chorionic villus core.

## Results from cohort studies

In contrast to experimental studies that indicate (partial) resistance to ZIKV of the second- and third-trimester placentas, the results of clinical cohort studies show that ZIKV-associated congenital abnormalities also occur in infants from mothers who had a ZIKV infection in the second or third trimester of pregnancy, albeit less frequently [10, 31, 32]. Preliminary data from the United States Zika Pregnancy Registry demonstrated that 8% of the infants from mothers who had laboratory-confirmed ZIKV infection during the first trimester of pregnancy had birth abnormalities, with 5% and 4% in the second and third trimesters, respectively [31]. A case-control study from Rio de Janeiro in 2016 reported that 55% of infants from mothers who were ZIKV PCR positive during the first trimester of pregnancy had birth abnormalities, compared with 52% and 29% during second and third trimesters [10]. Finally, a cohort study performed in French territories in the Americas found that ZIKV-related congenital abnormalities were present in 12.7% of the infants of women who had a PCR-confirmed, symptomatic ZIKV infection during the first trimester of pregnancy, whereas this was 3.6% and 5.3%, respectively, for the second and third trimesters [32].

In conclusion, there seems to be a discrepancy between the results from experimental research, which indicates that ZIKV cannot efficiently replicate in the protective trophoblasts of the term placenta, and data from clinical cohort studies, which demonstrate that the risk of congenital abnormalities is still significant when a ZIKV infection occurs in the third trimester of pregnancy. One explanation for this discrepancy could be the presence of a cofactor that enhances the ability to infect placental cells—for instance, the presence of cross-reactive dengue antibodies. This is a factor that is not accounted for in experimental research but that is present in a large part of the population in the clinical cohort studies from the Americas. In the next paragraphs, we will discuss how dengue antibodies can potentially exert a detrimental effect on infections, either via “traditional” ADE or through different mechanisms that make these antibodies a potential risk factor for ZIKV congenital abnormalities.

## ADE

Flavivirus antibodies pose a challenge for serological diagnostic tests, as they often bind not only to the virus a person was infected with but also to related flaviviruses. The presence of cross-reactive antibodies can also have a disease-enhancing effect via the principle of ADE. ADE of a flavivirus infection was first described in the 1960s, when it was observed that severe DENV infection occurred mainly during secondary infections and in infants that had subneutralizing levels of maternal antibodies, i.e., below the level needed to protect against a primary DENV infection [33]. It was hypothesized that antibodies resulting from infection with one DENV serotype might enhance disease in a subsequent infection with a different DENV serotype by a process called ADE [34]. According to the ADE hypothesis, antibodies produced during primary DENV infection can bind to a different DENV serotype but cannot neutralize it. These cross-reactive antibodies can facilitate the entry of the nonneutralized virus–antibody complexes (immune complexes), mainly via fragment crystallizable (Fc) gamma receptors (FcγRs), into the mononuclear phagocytic cells (MPCs). Antibody-mediated entry of virus in MPCs may result in either more infected cells (extrinsic ADE) or a more skewed T helper 2 (Th2) response (intrinsic ADE) [35, 36]. Infected MPCs may then serve as a reservoir to facilitate the viruses to reach different tissues in the body, resulting in more widespread infection, increased number of viral progeny, and worsening of disease [34, 37]. Different epidemiological studies have provided evidence that the incidence of severe DENV disease is higher among first-time-infected infants born to DENV-immune mothers and children who had developed a mild or asymptomatic dengue infection and became secondarily infected by a different DENV serotype [38, 39].

To test the hypothesis of ADE for DENV infections, a significant amount of experimental research has been performed [40–46]. Most in vitro studies have repeatedly reported FcγR-mediated enhancement of DENV infection in the presence of subneutralizing concentrations of cross-reacting antibodies against different DENV serotypes [40, 43, 47]. Mouse and nonhuman primate (NHP) models provided evidence for the potential clinical relevance of ADE for DENV. Mainly in mouse models, these studies showed increased viral loads and poor disease outcome during secondary DENV infection [42, 44–48].

Recently, results from a longitudinal cohort study with more than 6,000 children in Nicaragua confirmed that preexisting DENV antibodies were directly associated with disease severity in a dose-dependent manner [16]. Furthermore, in a follow-up study from phase III clinical trials for the live-attenuated tetravalent dengue vaccine Dengvaxia (CYD-TDV), it was observed that three years after the administration of this vaccine, the risk of hospitalization for DENV was increased in children younger than nine years of age [49, 50]. One of the hypotheses for this observation is that vaccine-related ADE of a subsequent DENV infection causes the increase in hospitalization for DENV in the vaccinated group of children younger than nine years old [51–53]. These observations provide evidence that preexisting nonneutralizing, binding DENV antibodies are an important risk factor for the occurrence of severe DENV disease. As DENV and ZIKV are closely related, the observations to date raise concerns about the impact of preexisting flavivirus immunity in determining the disease outcome for closely related and cocirculating flaviviruses, such as ZIKV.

## Evidence required for ADE of ZIKV

**Fig 1:**
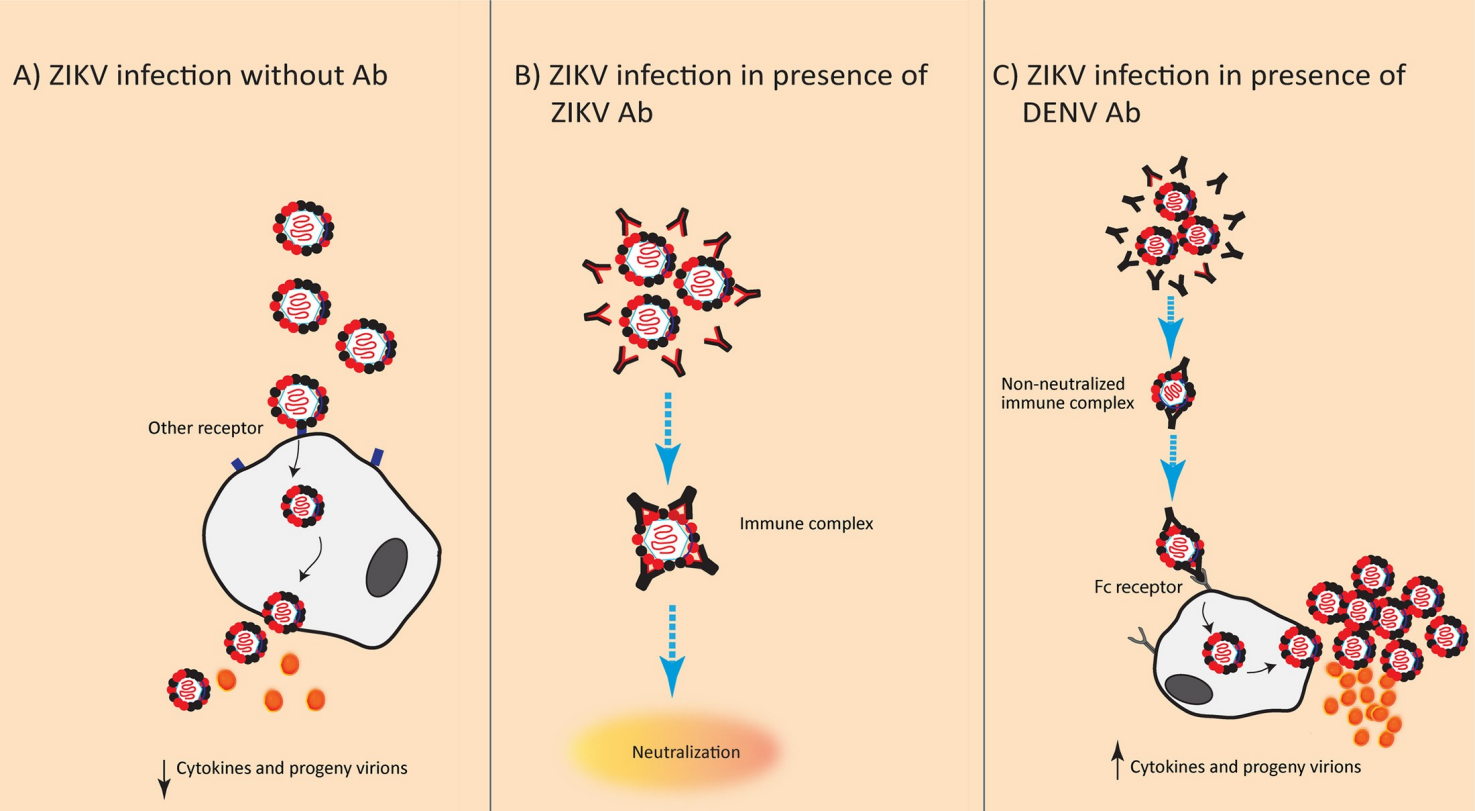
Proposed mechanism of ADE of ZIKV infection mediated by cross-reactive anti-DENV antibodies. (A) Primary ZIKV infection in naïve individuals. Entry occurs via other receptors and leads to virus and cytokine production. (B) Secondary ZIKV infection in a ZIKV-preimmune individual. Neutralization occurs effectively. (C) ZIKV ADE (black antibodies; preexisting antibodies against primary infecting DENV) Abs in immune sera can cross-react with ZIKV, allowing entry of the virus–antibody complexes into MPCs via the Fc receptor, leading to higher viral load along with higher levels of pro- and/or anti-inflammatory cytokines than cells infected in absence of antibodies. Ab, antibody; ADE, antibody-dependent enhancement; DENV, dengue virus; Fc, fragment crystallizable; MPC, mononuclear phagocytic cell; ZIKV, Zika virus.

Soon after the 2015–2016 ZIKV outbreak in the Americas, research was initiated to investigate whether ADE of a ZIKV infection could occur in the presence of flavivirus-reactive antibodies (notably, antibodies to DENV) and whether this could explain the seemingly increased pathogenicity of ZIKV, as proposed in Fig 1. Most of the results published so far are derived from experimental studies performed in myeloid cell lines or in animal models in which variables such as mortality, viremia, and proinflammatory cytokines are compared between flavivirus-preimmune and -naïve animals upon infection with ZIKV. As stated by Scott Halstead, one of the scientists who first described the ADE hypothesis, evidence that a microbial disease is worsened by ADE should not only come from experimental research, in which in situ replication of the causative organism in myeloid cells is demonstrated, but also come from epidemiological studies, as observations from animal experiments cannot always be extrapolated to the effects observed in infections in humans [54]. In the next paragraphs, an overview of the results from epidemiological and experimental research that studied ADE in ZIKV will be given.

## Need for epidemiological studies

Epidemiological studies investigating the occurrence of ADE of ZIKV infection are scarce, and epidemiological evidence for the traditional signs of ADE, such as an increased viral load or aberrant immune response leading to more severe disease, is currently lacking for ZIKV. Two epidemiological studies have determined the clinical outcomes of ZIKV infection in DENV-naïve and -preimmune patients [55, 56]. One of the studies did not find significant differences in cytokine profiles and ZIKV viremia in DENV-naïve and -preimmune patients [56]. Likewise, the other study also did not report any association between abnormal birth outcomes and preexisting DENV antibodies [55]. However, both of these studies had a small sample size and, therefore, had a low power for detecting differences in viral loads, cytokines, disease severity, and birth outcomes between the groups. For comparison, the recent publication providing convincing evidence for DENV ADE at the population level was based on a cohort of more than 6,000 individuals (15).

## Experimental studies: Contrary findings from in vitro and in vivo studies

In addition to epidemiological studies, several experimental studies using either preimmune sera/plasma or monoclonal antibodies (mAbs) have been conducted to investigate the enhancing role of flavivirus cross-reactive antibodies in ZIKV infection (Table 1).

**Table 1:** Overview of in vitro and in vivo studies investigating ADE in ZIKV infections.

Study	Preimmune sera/plasma mAbs		In vitro		In vivo	
			Cell line	ADE	Model	ADE
**Dejnirattisai and colleagues** [59]	DENV plasma	mAbs	U937	+		
**Swanstrom and colleagues** [68]		mAbs	U937	−	*IFNAR*^*−/−*^C57BL/6 mice	−
**Paul and colleagues** [60]	DENV sera	mAbs	K562	+		
**Priyamvada and colleagues** [61]	DENV sera	mAbs	U937	+		
**Stettler and colleagues** [67]	DENV	mAbs	K562	+	*AG129* mice	−
**Charles and colleagues** [58]		mAbs	THP1	+		
**Bardina and colleagues** [63]	DENV & WNV plasma		K562	+	*Stat2*^*−/−*^ C57BL/6 mice	+
**Castanha and colleagues** [57]	DENV serum	mAbs	K562	+		
**Slon Campos and colleagues** [62]	DENV vaccinated sera		K562	+		
**Kam and colleagues** [64]		mAb	K562	−	*IFNAR*^*−/−*^ mice	−
**Pantoja and colleagues** [66]	DENV sera		K562	+	Macaque	−
**Duehr and colleagues** [72]	TBEV sera		K562	+	*Stat2*^*−/−*^ mice	−
**McCracken and colleagues** [65]	DENV, YFV sera		U937, K562	+	Macaque	−

Abbreviations: ADE, antibody-dependent enhancement; *AG129 mice*, type I and II interferon receptor–lacking mice; DENV, dengue virus; *IFNAR*^*–/–*^, type I interferon receptor–lacking mice; mAb, monoclonal antibody; *Stat2*^*−/−*^, signal transducer and activator of transcription knockout mice; TBEV, tick-borne encephalitis virus; YFV, yellow fever virus.

In different in vitro studies, human DENV-immune plasma and/or a panel of DENV-specific human mAbs were used to determine the cross-reactivity as well as neutralizing and infection-enhancing properties of these antibodies against ZIKV infection [57–62]. Similar to the results of in vitro studies with DENV, enhanced ZIKV titers were detected in the presence of both DENV-preimmune sera and DENV-specific human mAbs by using FcγR-bearing human monocytic cell lines.

However, unlike in vitro studies, there are contrary findings in in vivo studies about the role of preexisting DENV antibodies in facilitating enhanced ZIKV pathogenesis [63–68]. In most of the studies conducted so far, enhanced ZIKV pathology due to preexisting DENV antibodies via ADE has not been observed, with only one exception [63]. Bardina and colleagues have reported an in vivo enhancement of ZIKV infection in mice, with increased morbidity and mortality in the presence of DENV and WNV human immune plasma [63]. This study also suggested that preexisting ZIKV cross-reacting antibodies can either be protective or can enhance pathogenesis depending on the concentrations of these antibodies, in line with the observations from DENV research [63]. A recent in vivo study using NHPs described that a ZIKV infection, 2.8 years post-DENV infection, did not produce any sign of ADE because of insignificant differences of viremia duration between ZIKV-infected naïve and DENV-preimmune NHPs [66], which is not in contrast with previous in vivo findings from DENV studies. The lack of clinical confirmation of the in vitro ADE results can be explained by multiple factors, such as the in vivo model used or the strain or serotype of primary infecting DENV and secondary infecting ZIKV. Another issue that could explain this is the different characteristics of antibodies binding to FcγR between humans and mice—i.e., distribution of immunoglobulin G (IgG) subclasses—and binding affinities of Fc to FcγR [69]. Some studies suggest that binding affinities of human IgG–Fc to mouse FcγR are lower than to human FcγR, which would make the translation of ADE results obtained in the mouse model with human serum difficult and uncertain [70]. However, more recent studies suggest that human IgG binds to mouse FcγR with similar affinities as to human FcγR [71]. Additionally, the duration between primary versus secondary infections, dose and route of infection, and titers and biological properties of cross-reactive IgG antibodies (such as IgG subclasses and, presumably, Fc glycosylation of these antibodies) can influence the outcome of ADE studies.

Unlike for DENV, the infection of MPCs by ZIKV-immune complexes has not been evaluated in vivo. Therefore, there is a need to determine whether the cell tropism of ZIKV infections differs between DENV-naïve and -preimmune individuals and to assess the potential for disease enhancement through properly powered epidemiological studies.

## Enhancement of DENV by ZIKV antibodies

**Fig 2:**
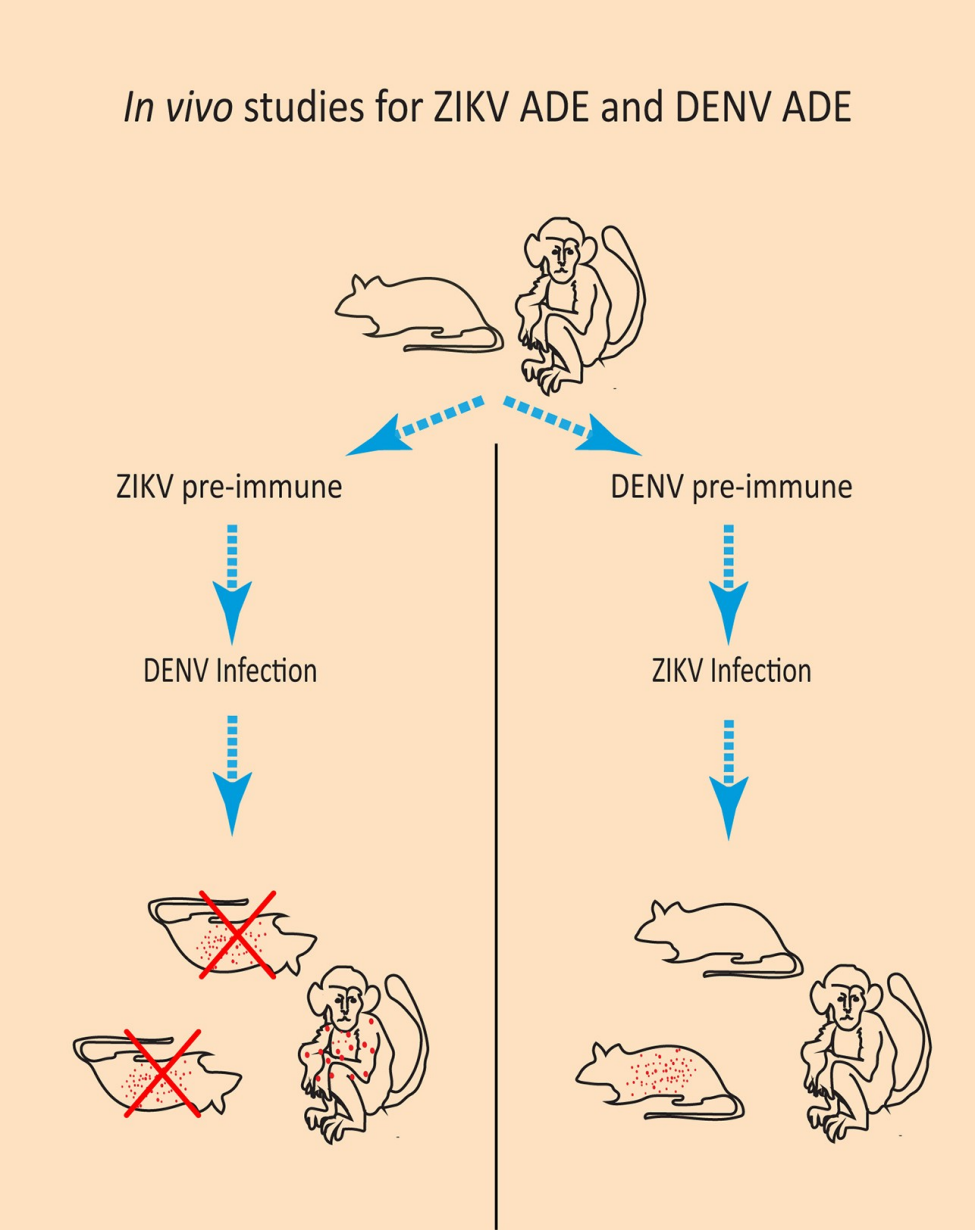
Results from in vivo studies investigating the role of ZIKV antibodies in DENV infection and on DENV antibodies in ZIKV infection. Left panel: In mice that had ZIKV antibodies either maternally acquired or administered before DENV infection, increased DENV viral load, cytokine production, and mortality was observed. In macaques that were previously infected with ZIKV, an increased DENV viral load but no clinical symptoms or mortality was observed upon infection with DENV. Right panel: In some DENV-preimmune mice that were infected with ZIKV, an increased viral load and cytokine production but not mortality was observed. In DENV-preimmune macaques infected with ZIKV, no changes in viral load, cytokine production, or mortality was observed in DENV. ADE, antibody-dependent enhancement; DENV, dengue virus; ZIKV, Zika virus.

Whereas most studies have focused on investigating the possibility of ZIKV ADE by DENV antibodies, ADE of DENV by preexisting ZIKV antibodies could be more clinically relevant. This is because of severe disease complications that are associated with DENV ADE, such as dengue hemorrhagic fever, dengue shock syndrome, and possibly also a worsened maternal and perinatal outcome when occurring during pregnancy [16, 73–77]. Two in vivo studies demonstrated more severe disease symptoms and mortality in DENV-infected mice that were pretreated with a ZIKV mAb or that had maternally acquired ZIKV antibodies compared with mice without ZIKV antibodies (Fig 2) [67, 78]. In a study with rhesus macaques, it was observed that the macaques that were previously infected with ZIKV had a significantly higher DENV viral load and proinflammatory cytokine production upon DENV-2 infection compared with ZIKV-naïve macaques [79]. However, no signs of dengue hemorrhagic fever were observed in these macaques; thus, only ADE of infection was observed, without changes in disease severity [79]. Overall, these studies indicate that prior ZIKV exposure might be a risk factor for DENV ADE. On the other hand, observations from arbovirus surveillance in Brazil suggest a decrease in DENV circulation after the ZIKV outbreak, possibly due to DENV cross-neutralization by ZIKV antibodies [80]. Additionally, there are indications that these cross-neutralizing ZIKV antibodies can prevent DENV ADE [81, 82]. However, for DENV, it is demonstrated that the risk of severe disease depends on the titer of preexisting DENV antibodies [16]. Therefore, it is plausible that cross-neutralizing ZIKV antibodies can prevent DENV ADE, whereas cross-reactive, binding ZIKV antibodies can enhance DENV infection, stressing the importance of measuring the balance between neutralizing and nonneutralizing antibodies in studies on pathogenesis [81]. The possibility of DENV ADE by ZIKV antibodies is especially of importance in DENV-naïve persons who live in DENV-endemic areas and who have had a previous ZIKV infection. Furthermore, the possibility of ZIKV vaccine–induced ADE of a DENV infection should be taken into account for the evaluation of a future ZIKV vaccine.

## The role of cross-reactive antibodies in ZIKV-associated congenital abnormalities

The literature discussed thus far has focused on addressing the possibility of enhancement of ZIKV disease in an infected person with prior DENV exposure. However, an important question is whether—rather than the “DENV” mechanism of ADE, which focuses on cytokine production, viral load, or mortality—the clinical presentation of ZIKV infection enhancement by cross-reactive antibodies might be missed because ZIKV has a broader tissue tropism than DENV and can be detected in, among others, the placenta, the reproductive tract, the eyes, and brain tissue [17, 18]. Even though there are no reports of worsened ZIKV disease in individuals with prior DENV exposure, is it possible that cross-reactive flavivirus antibodies can still be a risk factor for the ZIKV-associated congenital anomalies?

## Neonatal Fc receptor–mediated transcytosis across the placenta

From weeks 20–24 of pregnancy, when the placenta is fully developed, maternal IgG antibodies are actively transported across the placenta from mother to fetus through neonatal Fc receptor (FcRn)-mediated transcytosis in STBs [83–85]. STBs internalize fluid containing maternal IgG at the apical surface; the Fc region of IgG can subsequently bind the FcRn in acidic endosomes, after which IgG is released at the basolateral surface at a neutral pH [86]. The hypothesis that transcytosis of IgG–virion complexes across the placenta can occur has been confirmed in in vitro studies that demonstrated that IgG–virion complexes of human immunodeficiency virus (HIV) and CMV can be transcytosed across FcRn-bearing epithelial cells and that this process can be inhibited or completely blocked when the FcRn is blocked or knocked down [87, 88]. In an ex vivo study using placental explants, it was demonstrated that CMV could be transcytosed across the STB layer in the presence of both high and low neutralizing antibodies [88]. However, in the presence of high neutralizing antibodies, CMV virions were captured by villus core macrophages and were unable to replicate, whereas in the presence of low neutralizing antibodies, viral replication was detected in CTB progenitors beneath an intact and uninfected STB layer [88].

**Fig 3:**
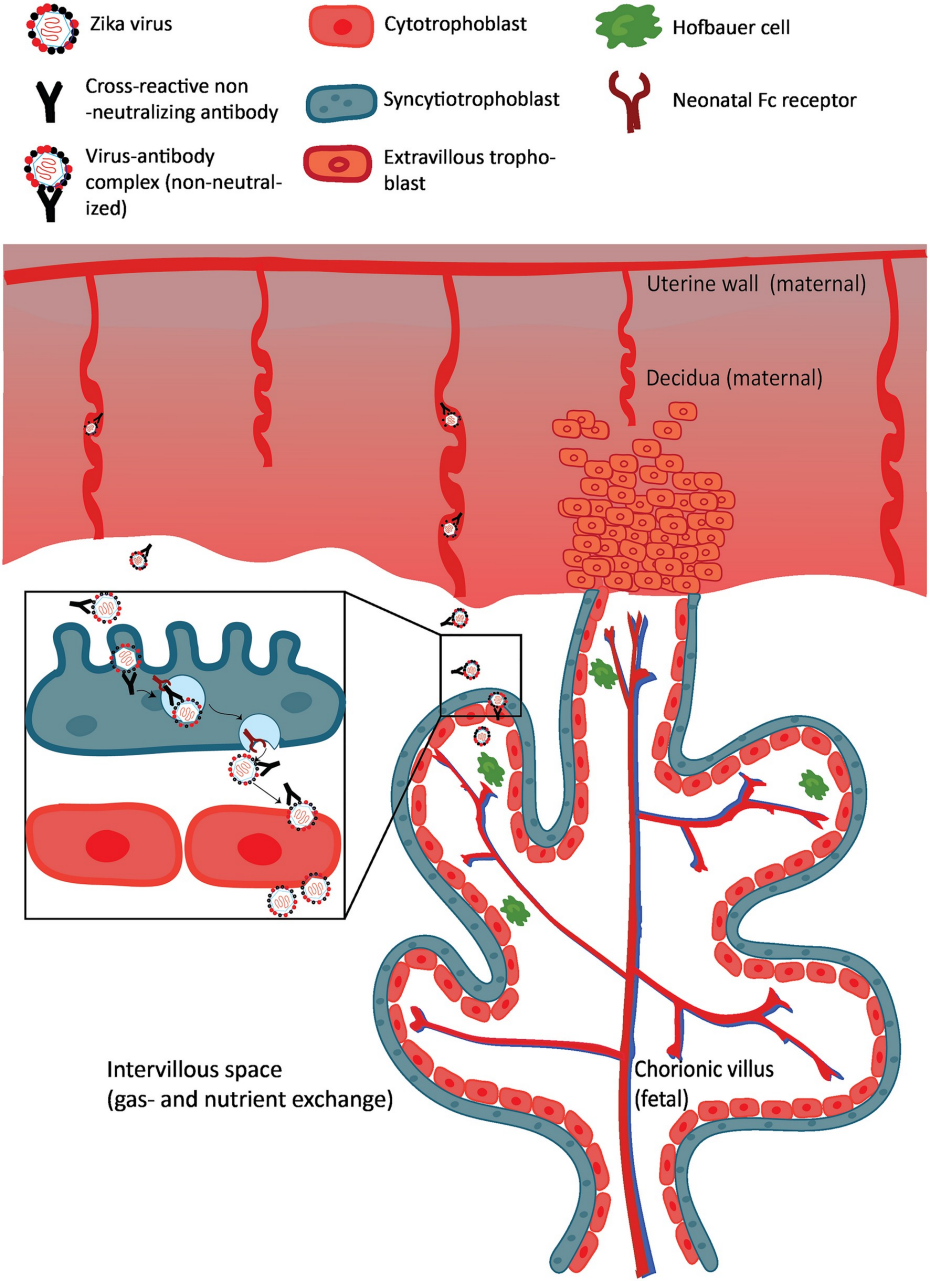
Proposed mechanism of FcRn-mediated transcytosis of a ZIKV–IgG complex in a chorionic villus. Illustrated is a chorionic villus that is anchored to the mucosal lining of the uterus (decidua). Through the circulation of the mother, ZIKV bound to maternal cross-reactive flavivirus IgG antibodies is present in the intervillous space. This IgG–virion complex can subsequently cross the syncytiotrophoblasts via FcRn-mediated transcytosis. When ZIKV is transcytosed across this trophoblast layer, it can infect the perivascular-located Hofbauer cells, after which viral progeny can cross the endothelial cell barrier, possibly with help from ZIKV NS1 protein, and reach the fetal circulation. FcRn, neonatal fragment crystallizable receptor; IgG, immunoglobulin G; NS1, nonstructural protein 1; ZIKV, Zika virus.

If this FcRn-mediated transcytosis is possible for ZIKV, Zika virions bound to maternal nonneutralizing, cross-reactive flavivirus antibodies could still be infective when released at the fetal side of the chorionic villus, similar to what has been found for CMV–IgG complexes (Fig 3). Once in the chorionic villus, ZIKV will encounter, among others, CTBs and HBCs. Because ZIKV can readily replicate in the perivascular-located HBCs, ZIKV could disseminate from HBCs to the fetal capillaries and enter the fetal circulation [27, 29, 89, 90]. A recent experimental study found indications that ZIKV can cross the trophoblast layer of the placenta through FcRn-mediated transcytosis. In this study, second-trimester placental explants were used to demonstrate that ZIKV infection of these explants was higher when ZIKV was preincubated with cross-reactive DENV mAbs, mainly IgG1 and IgG3 subclasses, compared with nonspecific influenza mAbs [91]. Blocking of the FcRn with an FcRn-specific mAb inhibited ZIKV replication by 16.5-fold [91]. The finding that ZIKV can infect placental explants more efficiently in the presence of DENV antibodies was confirmed by another recent study [92]. In this study, there was no enhancement of infection observed in the placental explants when ZIKV was preincubated with sera containing YFV or chikungunya virus antibodies, but in the presence of DENV antibodies, there was faster ZIKV replication and more virus production compared with the absence of DENV antibodies [92]. Furthermore, the clinical observation that, in several placentas of ZIKV infected women, ZIKV is only detected in HBCs and not in the trophoblasts lining the chorionic villi is another indication that transplacental FcRn-mediated transcytosis of ZIKV can occur in ZIKV-infected pregnant women [23, 24].

## Conclusion

The hypothesis that antibodies produced during a primary DENV infection may cause severe secondary DENV infection (through ADE) has been controversial for a long time. To date, the theory of ADE in DENV infection is more broadly accepted, mainly because a large epidemiological study provided clear evidence for enhanced risk of DENV complications in children with a specific range of preexisting antibodies. Studies performed to determine ZIKV ADE so far have found evidence for ADE in vitro, but compelling evidence in vivo is lacking, whereas ADE of a DENV infection in the presence of cross-reactive ZIKV antibodies is observed in several in vivo studies. Based on the current literature, there is not enough evidence to confirm or disprove definitively that the ADE observed in vitro plays an important role in ZIKV pathogenicity. It is unlikely that ADE of a ZIKV infection in humans would result in the same disease complications as seen in DENV, as current studies have not found any indications of this effect. However, there is a less-well-researched possibility that cross-reactive flavivirus antibodies can cause other detrimental effects in ZIKV infection, possibly by facilitating transplacental transmission through FcRn-mediated transcytosis. Currently, properly designed clinical studies that find strong associations of cross-reactive flavivirus antibodies and congenital syndrome are missing. Therefore, large longitudinal cohort studies with pregnant women in flavivirus-endemic areas are needed to assess the potential role of cross-reactive flavivirus antibodies in pathogenesis of fetal infection and disease when a ZIKV infection occurs during pregnancy. For these studies, serological discrimination of cross-reactive flavivirus antibodies will be crucial. Fundamental knowledge of the pathogenesis of this severe illness remains important, particularly in light of potential consequences for flavivirus vaccination.
